# Familial Coaggregation of Asthma and Type 1 Diabetes in Children

**DOI:** 10.1001/jamanetworkopen.2020.0834

**Published:** 2020-03-12

**Authors:** Awad I. Smew, Cecilia Lundholm, Lars Sävendahl, Paul Lichtenstein, Catarina Almqvist

**Affiliations:** 1 Department of Medical Epidemiology and Biostatistics, Karolinska Institutet, Stockholm, Sweden; 2 Pediatric Endocrinology Unit at Astrid Lindgren Children’s Hospital, Karolinska University Hospital, Stockholm, Sweden; 3 Pediatric Allergy and Pulmonology Unit at Astrid Lindgren Children’s Hospital, Karolinska University Hospital, Stockholm, Sweden

## Abstract

**Importance:**

The association between atopic and autoimmune disease, particularly asthma and type 1 diabetes, has been debated. Further understanding of the underlying factors associated with the comorbidity in children is warranted.

**Objectives:**

To assess the bidirectional association between asthma and type 1 diabetes and examine the possibility of a shared risk for the diseases by studying their pattern of familial coaggregation.

**Design, Setting, and Participants:**

A birth cohort study of children born from January 1, 2001, and followed up until December 31, 2015, was performed. Population data were obtained from multiple national Swedish registers. A total of 1 347 901 singleton children, live-born in Sweden between January 1, 2001, and December 31, 2013, were identified, and children with incomplete data were excluded. The remaining 1 284 748 children were linked to their biological full siblings, maternal and paternal half-siblings, cousins, and half-cousins. Data analysis was conducted from April 1, 2019, to January 17, 2020.

**Main Outcomes and Measures:**

Cases of asthma and type 1 diabetes were defined using a combination of diagnoses and medication prescriptions found in the registers.

**Results:**

In the cohort of 1 284 748 children, 660 738 children (51.4%) were boys; 121 809 children (9.5%) had asthma, 3812 children (0.3%) had type 1 diabetes, and 494 children had both asthma and type 1 diabetes, representing 0.4% of all asthma or 13% of all type 1 diabetes. Mean (SD) age at diagnosis was 3.0 (2.8) years for children with asthma, and 5.9 (3.3) years for those with type 1 diabetes. Asthma and type 1 diabetes were associated within individuals (odds ratio, 1.15; 95% CI, 1.05-1.27). Children with asthma had an increased risk of subsequent type 1 diabetes (hazard ratio, 1.16; 95% CI, 1.06-1.27); however, subsequent asthma risk did not differ substantially among children with type 1 diabetes (hazard ratio, 0.92; 95% CI, 0.75-1.12). Siblings of individuals with asthma were at an increased risk of type 1 diabetes (odds ratio, 1.27; 95% CI, 1.13-1.42) and vice versa. The results remained positive after controlling for the direct association of one disease with the other.

**Conclusions and Relevance:**

This study appears to provide evidence for co-occurrence, importance of sequential appearance, and coaggregation of asthma and type 1 diabetes in children and their siblings. The findings may suggest shared familial factors contributing to the associations. Knowledge of the nature of the association could be of importance in future clinical practice.

## Key Points

**Question:**

Is there an association between childhood asthma and type 1 diabetes and do shared familial factors contribute to the comorbidity?

**Findings:**

In this cohort study including data from 1 284 748 children born in Sweden, asthma and type 1 diabetes co-occurred in individuals and coaggregated within families. Siblings of children with one disease were themselves at an increased risk of the other disease, suggesting a shared familial liability.

**Meaning:**

Knowledge of the comorbidity between asthma and type 1 diabetes and familial coaggregation extending to siblings is important in understanding the association between atopic and autoimmune disease and may be of future clinical importance.

## Teaser

This population-based cohort study examines the co-occurrence of asthma and type 1 diabetes, as well as familial aggregation of the diseases, in children.

## Introduction

Asthma is globally the most prevalent chronic childhood disease affecting approximately 11% of children aged 6 to 7 years.^1^ Type 1 diabetes is one of the most common childhood autoimmune diseases, with the incidence rates in Sweden among the highest worldwide.^2^ Previous studies have demonstrated positive associations between asthma and type 1 diabetes,^3,4,5,6,7,8,9,10,11^ indicating their concomitant occurrence at a population level and individual level and presenting evidence of the importance of the sequential appearance of disease.^7^ However, results have been conflicting, and negative associations have also been shown.^12,13^ The range of findings could be explained by heterogeneous methods, including various study designs, inclusion of both children and adults, lack of power, and differing definitions of disease.

The mechanisms that factor in to the association between asthma and type 1 diabetes are still unclear. Findings of co-occurrence of the diseases within individuals are in direct opposition to the previously proposed mutual inhibition of helper T cell T_h_1- and T_h_2-mediated immune responses.^11^ Instead, more complex mechanisms involving T_h_17 cells and regulatory T cells have been implicated.^14^ In favor of the positive association between asthma and type 1 diabetes is their similar increases in incidences over the past decades.^15^ This similarity could suggest a common cause owing to changes in environmental factors, such as microbial exposure, socioeconomic status, geographic location during upbringing, and diet.^15,16,17^

Shared genetic factors may also exist. Genetic studies have shown certain overlaps in susceptibility loci between asthma and type 1 diabetes,^18^ although, to our knowledge, systematic comparisons of results from genome-wide association studies of asthma and type 1 diabetes have not yet been performed. Furthermore, asthma^19,20^ and type 1 diabetes^21,22,23^ are highly heritable diseases. Although heritability studies confirm an independent aggregation of these diseases in families, evidence is lacking for their familial coaggregation. Studying the familial coaggregation of asthma and type 1 diabetes among various types of relatives could help to explain the association between the diseases by providing evidence for the existence of etiologic factors—genetic, environmental, or both—shared within families.^24^

In a large nationwide study, we aimed to assess bidirectional associations between asthma and type 1 diabetes to aid in understanding the co-occurrence of the diseases and importance of their sequential appearance, as well as examine their familial coaggregation using a genetically informative design. We hypothesized that relatives of individuals with one disease would be at an increased risk for the other disease, indicating a shared familial component.

## Methods

**Figure 1.**
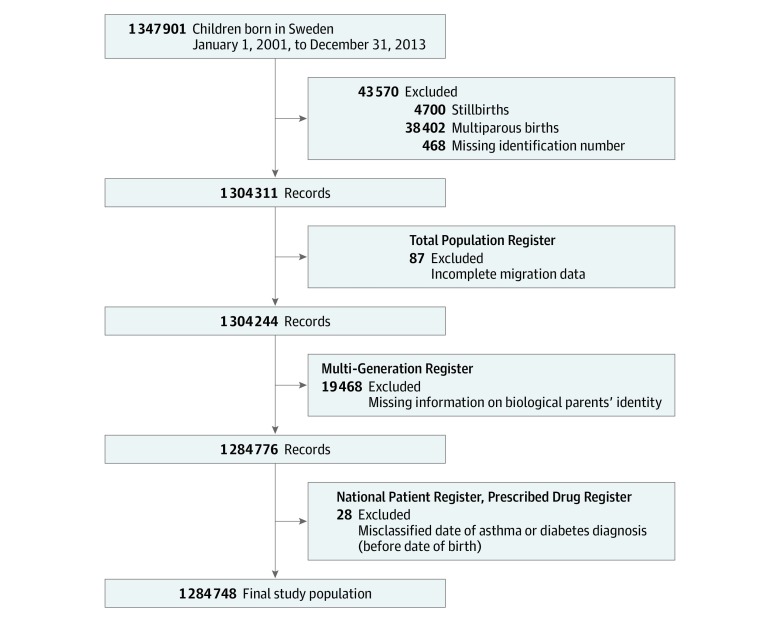
Description of the Study Population Children born in Sweden between January 1, 2001, and December 31, 2013, were identified from the Medical Birth Register. Only live-born, singleton children were included, and children with missing data from various Swedish national registers were excluded.

We conducted a population-based cohort study using data from multiple Swedish registers held by the National Board of Health & Welfare and Statistics Sweden, linked via the Swedish personal identity number.^25^ Singleton children, live-born in Sweden between January 1, 2001, and December 31, 2013, were identified from the Medical Birth Register, and children with missing data were excluded (Figure 1). Data on emigration and death were obtained from the Total Population Register.^26^ Using the Multi-Generation Register,^27^ we linked children to their biological parents. From this linkage, we identified all siblings, maternal half-siblings, paternal half-siblings, cousins, and half-cousins within the cohort, creating 5 subcohorts. Each subcohort included all possible relative pairs; that is, each individual contributed at least twice—once as an index individual and once as a relative.

Ethical approval for this study was granted by the regional ethical review board in Stockholm, Sweden. The board waived the requirement of informed consent owing to the register-based study design and use of deidentified personal information. This study followed the Strengthening the Reporting of Observational Studies in Epidemiology (STROBE) reporting guideline for cohort studies.

Definitions of asthma and type 1 diabetes were based on a combination of diagnoses and dispensed medication prescriptions from the National Patient Register and the Prescribed Drug Register (eMethods in the Supplement).

### Statistical Analysis

First, a conditional logistic regression was used to assess the association between any diagnosis of asthma and type 1 diabetes at the end of the follow-up period. This model, conditioned on date of birth, assessed the risk, which is presented as crude and sex- and date of birth–adjusted odds ratios (ORs) and 95% CIs, of asthma at the end of follow-up among individuals with type 1 diabetes compared with individuals without type 1 diabetes or vice versa.

Second, to avoid differences in follow-up time, the associations between the cumulative incidences of the 2 diseases at different ages (5, 6, 7, or 8 years) were examined separately using logistic regression, calculating both crude and sex- and date of birth–adjusted ORs and 95% CIs. Each disease was assessed as both a dependent and independent variable. We present the model using type 1 diabetes as the independent variable and asthma as the dependent variable, but the results are bidirectional. Because we studied cumulative incidence cross-sectionally at the end of follow-up and at specific ages, one disease did not necessarily have to occur before the other disease.

Third, the respective associations between previous asthma and the risk of subsequent type 1 diabetes and between previous type 1 diabetes and the risk of subsequent asthma were estimated separately using Cox proportional hazards regression, with age as the underlying timescale and previous disease modeled as a time-varying independent variable. Follow-up started at birth and individuals were censored at emigration, death, or December 31, 2013, for children younger than 4.5 years or December 31, 2015, for children 4.5 years or older on December 31, 2013, owing to differences in asthma definition depending on age (eMethods in the Supplement), whichever occurred first. The assumption of proportional hazards was tested based on Schoenfeld residuals. We found no evidence of nonproportional hazards and therefore did not explore any age-varying associations. Hazard ratios (HRs) were adjusted for sex and date of birth. Because we were interested in estimating within-individual associations, despite the presence of possible familial factors, to understand their co-occurrence rather than causal relationship, no other covariates were adjusted for.

To examine the familial coaggregation of asthma and type 1 diabetes, logistic regression was applied in each subcohort of relatives with models estimating the risk (as OR) of asthma (dependent variable) in relatives of individuals with type 1 diabetes (independent variable) compared with the risk of asthma in relatives of individuals without type 1 diabetes, and vice versa.

Because relatives share a higher number of genetic and environmental factors compared with nonrelatives, a higher risk of one disease in relatives of individuals with another disease compared with relatives of individuals without that other disease may point to evidence for shared etiologic factors within families associated with the co-occurrence of both diseases. Furthermore, differences in risk between relatives of varying genetic and environmental relatedness may help in understanding the type of shared familial factors in the association. For example, if an association was mainly a consequence of shared genetic factors, the association would decrease with increasing genetic distance between relatives.

In addition, each model was adjusted for sex and date of birth of the relative as well as for the possibility of a direct association between one disease and the other, ie, adjusted for asthma in the relative when estimating risk of type 1 diabetes in the relative and vice versa. Associations that remain positive even after adjusting for direct effects may give further support of the shared familial factors to both diseases.^24,28^

All within-individual and familial coaggregation analyses for full siblings were repeated in sensitivity analyses. Stricter definitions of type 1 diabetes were used to exclude children with onset of disease when they were younger than 1 year, as well as identifying children with type 1 diabetes based separately on either diagnosis or insulin prescription. The analyses were also applied to a restricted cohort of children born between January 1, 2005, and December 31, 2013, to avoid left truncation owing to the Prescribed Drug Register starting in 2005.

In all statistical analyses, we used 2-sided 5% significance levels. To correct for nonindependence owing to familial clustering, the sandwich estimator for SEs was applied. Data management was performed using SAS, version 9.4 (SAS Institute Inc) and analyses using Stata, version 15.1 (StataCorp), from April 1, 2019, to January 17, 2020.

## Results

Our study population (Figure 1) consisted of 1 284 748 children (660 738 boys [51.4%] and 624 010 girls [48.6%]). In the cohort, 121 809 children (9.5%) had asthma and 3812 children (0.3%) had type 1 diabetes. Mean (SD) age at diagnosis was 3.0 (2.8) years for asthma and 5.9 (3.3) years for type 1 diabetes. In total, we identified 494 children with both asthma and type 1 diabetes, representing 0.4% of all asthma or 13.0% of all type 1 diabetes (Table 1). We also identified 835 412 full siblings (in 1 083 788 full sibling pairs) with similar prevalence of disease (9.6% of children with asthma, 0.3% with type 1 diabetes, and 0.04% with both). Further descriptive statistics for the 5 relative subcohorts are presented in eTable 1 in the Supplement.

**Table 1.** Child Characteristics by Asthma and Type 1 Diabetes Status

Characteristic	Full Cohort	Asthma	Type 1 Diabetes	Asthma and Type 1 Diabetes
Total No. of children (%)	1 284 748	121 809 (9.5)	3812 (0.3)	494 (0.04)
Age at first diagnosis, mean (SD), y				
Asthma	NA	3.0 (2.8)	NA	3.4 (3.0)
Type 1 diabetes	NA	NA	5.9 (3.3)	6.2 (3.3)
Sex, %				
Male	51.4	61.8	51.7	60.5
Female	48.6	38.2	48.3	39.5

Abbreviation: NA, not applicable.

Asthma and type 1 diabetes at the end of the follow-up period were positively associated (OR, 1.15; 95% CI, 1.05-1.27). Examination of the cumulative incidence of disease at specific ages showed ORs ranging from 1.33 (95% CI, 1.13-1.56) at age 5 years to 1.44 (95% CI, 1.28-1.63) at age 8 years (Table 2).

**Table 2.** Within-Individual Analyses of the Association Between Asthma and Type 1 Diabetes in Children Born From 2001 to 2013, Assessed at End of Follow-up and at Different Ages

End of Follow-up^a^	Type 1 Diabetes		No Type 1 Diabetes		OR (95% CI)	
	Total, No.	Asthma, No. (%)	Total, No.	Asthma, No. (%)	Crude	Adjusted^b^
Overall	3812	494 (13.0)	1 280 936	121 315 (9.5)	1.15 (1.05-1.27)	1.15 (1.05-1.27)
Age 5 y	1479	172 (11.6)	953 123	85 870 (9.0)	1.33 (1.13-1.56)	1.32 (1.12-1.55)
Age 6 y	1738	227 (13.1)	843 877	83 647 (9.9)	1.37 (1.19-1.57)	1.36 (1.18-1.56)
Age 7 y	1918	277 (14.4)	740 370	77 485 (10.5)	1.44 (1.27-1.64)	1.44 (1.26-1.63)
Age 8 y	2057	309 (15.0)	639 059	69 859 (10.9)	1.44 (1.28-1.63)	1.42 (1.26-1.60)

Abbreviation: OR, odds ratio.

^a^ Age at end of follow-up ranged from 1 day to 15 years.

^b^ Analyses adjusted for sex and date of birth.

Of 121 390 children with asthma and no previous diagnosis of diabetes, 394 children (0.3%) subsequently developed type 1 diabetes. Of 3073 children with type 1 diabetes and no previous diagnosis of asthma, 97 children (3.2%) subsequently developed asthma. We found a positive association for risk of subsequent type 1 diabetes following a previous diagnosis of asthma (HR, 1.16; 95% CI, 1.06-1.27). In contrast, type 1 diabetes was not associated with risk of subsequent asthma (HR, 0.92; 95% CI, 0.75-1.12) (Table 3).

**Table 3.** Bidirectional Within-Individual Associations of the Risk of Subsequent Disease (Asthma or Type 1 Diabetes) If Previous Exposure to the Other Disease (Type 1 Diabetes or Asthma) in Children Born From 2001 to 2013^a^

Variable	Total, No.	Disease, No. (%)		Length of Follow-up, Mean (SD), y	HR (95% CI)	
		Previous	Subsequent^b^		Crude	Adjusted^c^
Subsequent asthma if previous type 1 diabetes	1 284 748	3073 (0.2)	97 (3.2)	6.6 (4.6)	0.92 (0.75-1.12)	0.91 (0.75-1.11)
Subsequent type 1 diabetes if previous asthma	1 284 748	121 390 (9.4)	394 (0.3)	7.4 (4.1)	1.16 (1.06-1.27)	1.17 (1.07-1.28)

Abbreviation: HR, hazard ratio.

^a^ Followed up from birth until disease onset, death, emigration, or December 31, 2015, whichever occurred first, using Cox proportional hazards regression.

^b^ The number of individuals receiving subsequent diagnosis of a second disease among those individuals with the first disease and no previous diagnosis of the second.

^c^ Analyses adjusted for sex and date of birth.

**Figure 2.**
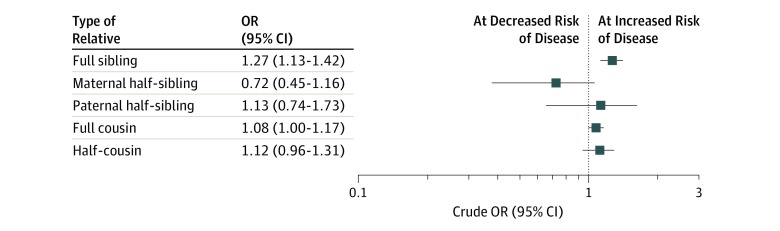
Familial Coaggregation of Asthma and Type 1 Diabetes Crude odds ratios (ORs) represent the bidirectional association between asthma and type 1 diabetes across different types of relatives, and actual values are displayed on a logarithmic scale. Full data for familial coaggregation analyses are found in eTable 2 in the Supplement.

Full siblings of individuals with one disease were at an increased risk of the other disease (OR, 1.27; 95% CI, 1.13-1.42) (Figure 2). The coaggregation in full cousins was also positive, yet attenuated (OR, 1.08; 95% CI, 1.00-1.17). No significant ORs were detected for half-siblings (OR, 0.72; 95% CI, 0.45-1.16 for maternal half-siblings and OR, 1.13 95% CI, 0.74-1.73 for paternal half-siblings) or half-cousins (OR, 1.12; 95% CI, 0.96-1.31).

After additional adjustment for the direct association of one disease with the other, results for full siblings remained positive. Siblings of individuals with type 1 diabetes were at a higher risk of asthma even after adjusting for their own type 1 diabetes (OR, 1.25; 95% CI, 1.12-1.40), and uniformly, siblings of individuals with asthma were at a higher risk of type 1 diabetes even after adjusting for their own asthma (OR, 1.21; 95% CI, 1.08-1.36). Full data for all relative subcohorts are presented in eTable 2 in the Supplement.

Results of the within-individual analyses were similar in the sensitivity analyses either excluding children diagnosed with type 1 diabetes before age 1 year (n = 84) or defining type 1 diabetes based on diagnosis (n = 2935) or insulin prescription (n = 3597) separately (eTable 3 in the Supplement). Associations were stronger in the restricted cohort (n = 871 521) (eTable 4 in the Supplement). For the familial coaggregation analyses, the associations were unchanged using alternative type 1 diabetes definitions and remained positive yet attenuated in the restricted cohort (eTable 5 in the Supplement).

## Discussion

In this nationwide cohort study, we found associations between childhood asthma and type 1 diabetes both within individuals and families. We demonstrated a bidirectional association showing the co-occurrence of the 2 diseases and the apparent importance of the sequential appearance of disease, in which a previous diagnosis of asthma was associated with an increased risk of subsequent type 1 diabetes, whereas type 1 diabetes was not associated with an increased risk of subsequent asthma in children. The study also provides data on familial coaggregation, with siblings of individuals with asthma or type 1 diabetes at an increased risk of either disease, suggesting a shared familial risk.

The results of the bidirectional association between asthma and type 1 diabetes are in line with previously published findings,^3,4,5,6,7,8,9,10,11^ supporting the possible co-occurrence of both diseases in a large population-based sample.

In assessing risk of subsequent disease after previous diagnosis of the other disease, similarly to a recent Finnish population-based study of 81 473 asthma cases and 9541 type 1 diabetes cases,^7^ the direction of the association between asthma and type 1 diabetes appears to depend on the sequential development of disease. Metsälä et al^7^ also noted that previous diagnosis of asthma appeared to be associated with increased risk of subsequent type 1 diabetes (HR, 1.45; 95% CI, 1.32-1.60). However, their findings of decreased subsequent asthma risk after previous diagnosis of type 1 diabetes (HR, 0.70; 95% CI, 0.59-0.84) are in contrast to the lack of difference in asthma risk found in our study.

Our findings of an increased risk of type 1 diabetes after previous asthma diagnosis are consistent with results reported elsewhere.^5,8^ Surveillance bias probably does not explain these results given that children diagnosed with type 1 diabetes often present with acute symptoms and are swiftly referred for hospital treatment, even if not monitored for other conditions. However, we cannot reject that there may exist a causal pathway between asthma and development of type 1 diabetes, perhaps mediated via inhaled corticosteroid therapy.^29^

In parallel with our findings, other studies have not been able to detect differences in asthma risk after previous type 1 diabetes diagnosis.^12^ Some have, however, shown an increased subsequent risk of asthma,^4,6^ which could result from surveillance bias. In our results, even though type 1 diabetes did not appear to be associated with an increased risk of subsequent asthma, the risk of both diseases co-occurring in one individual was increased. Our findings could partially be explained by the fact that asthma in our sample occurred at a younger mean age than type 1 diabetes, meaning that children with both asthma and type 1 diabetes in general already had received their asthma diagnosis before they developed type 1 diabetes.

In familial analyses, we found that siblings of individuals with asthma appeared to be at an increased risk of type 1 diabetes, and vice versa. The results remained positive even after adjustment for the direct association of one disease with the other, thus strengthening the evidence of shared familial factors—genetic, environmental, or both—in the co-occurrence. In favor of a shared genetic source of the diseases is our finding that siblings of individuals with asthma were at a higher increased risk of type 1 diabetes and vice versa, compared with cousins, suggesting a diminishing risk with increasing genetic difference.

A previous study^30^ found associations between type 1 diabetes in children and a number of immune-mediated conditions, including asthma, in their parents (standardized incidence ratio, 1.31; 95% CI, 1.19-1.44) and siblings (standardized incidence ratio, 1.22; 95% CI, 0.79-1.87). The findings of coaggregation among first-degree relatives are similar to ours, given that parents and siblings share approximately the same amount of segregating genes (50%). However, the investigators of that study defined disease based only on hospital diagnoses, which may limit the identification of patients with asthma, given that only patients with the most severe disease are hospitalized.

From a research perspective, future studies using other genetically informative designs will be instrumental in further understanding of shared familial factors contributing to asthma and type 1 diabetes, for instance, through estimating heritability of the comorbidity using quantitative genetic modeling and/or linkage disequilibrium score regressions.

From a clinical perspective, although more evidence is needed before implementation of guidelines, awareness of the known association between the 2 diseases is important for physicians treating those patients. An understanding of the comorbidity could be beneficial in terms of avoiding diagnostic delay by recognizing symptoms of asthma that may otherwise be overlooked by caregivers and physicians in the more acute management of patients with type 1 diabetes.

### Strengths and Limitations

A main strength of this study is the large population-based sample with data recorded prospectively and originating from reliable Swedish registers,^26^ thereby increasing the generalizability of our findings to similar populations and eliminating the risk of recall bias. Furthermore, we used validated measures of disease, previously shown for both asthma^31^ and definition of type 1 diabetes using insulin prescription.^2^ In addition, the genetically informative design based on the identification and linkage of 5 types of relatives within our cohort allowed us to suggest familial coaggregation, thereby exploring the possibility of shared familial risk factors underlying the association between asthma and type 1 diabetes.

Despite these strengths, our results must be interpreted in light of several limitations. First, given data availability, our study may have right censoring reflected by the lower mean age at diagnosis and prevalence of type 1 diabetes in our cohort compared with other Swedish register studies.^32^ Nevertheless, the incidence of type 1 diabetes is increasing in younger children^33^ and we were able to examine the risk of disease at the specific younger ages, thereby contributing to the understanding of early-onset type 1 diabetes. Because administrative censoring is noninformative, it ought not to bias the results.

Second, the National Patient Register does not contain information on diagnoses from primary care and the Prescribed Drug Register only reports medication dispensed from 2005, which increases the risk of misclassification of milder asthma. The lack of these data may explain differences in the results of sensitivity analyses in the restricted cohort born from 2005. However, misclassification of type 1 diabetes based on diagnosis ought to be minimal because all children with type 1 diabetes are initially hospitalized, then routinely followed up in specialist outpatient clinics. Inclusion of other, rarer forms of diabetes in children, such as type 2 diabetes, is limited because type 1 diabetes is present in more than 98% of individuals in Sweden younger than 20 years who have diabetes.^34^ In addition, results excluding children diagnosed before age 1 year indicated that possible misclassification of neonatal diabetes did not bias the results.

Third, despite our large sample, the study was underpowered to detect differences in risk among half-siblings and half-cousins, reflected in the small number of cases and wide CIs for the estimates. Owing to register coverage, we were not able to assess patterns of disease in the parents of the children in the cohort.

## Conclusions

This population-based cohort study of more than 1 million Swedish children found evidence supporting co-occurrence of asthma and type 1 diabetes in individuals, importance of sequential appearance of disease, and familial coaggregation. These results indicate both an oversimplification of the T_h_1/T_h_2 paradigm and support evidence for a familial risk due to shared factors, despite the possible existence of causal pathways between the 2 diseases. These findings represent an important step in further understanding the nature of the association between atopic and autoimmune disease and may be of importance in the future clinical management of these patients.

## Notes

Supplement. **eMethods.** Detailed Methods **eReferences.** **eTable 1.** Asthma and Type 1 Diabetes in Each Sub-Cohort of Relatives **eTable 2.** Family-Level Bidirectional Associations Between Asthma and Type 1 Diabetes Across Different Types of Relatives in Children Born 2001-2013 **eTable 3.** Sensitivity Analyses for Within-Individual Analyses of the Associations Between Asthma and Type 1 Diabetes in Children Born 2001-2013, Assessed at End of Follow-Up and at Different Ages **eTable 4.** Sensitivity Analyses for Bidirectional Within-Individual Associations of the Risk (as Hazard Ratio) of Subsequent Disease (Asthma/Type 1 Diabetes) if Previous Exposure to the Other Disease (Type 1 Diabetes /Asthma) in Children Born 2001-2013, Followed From Birth Until Disease Onset, Death, Emigration, or 31st December 2015, Whichever Occurred First **eTable 5.** Sensitivity Analyses for Family-Level Bidirectional Associations Between Asthma and Type 1 Diabetes Across Full Siblings

## References

[1] Pearce N, Aït-Khaled N, Beasley R, ; ISAAC Phase Three Study Group Worldwide trends in the prevalence of asthma symptoms: phase III of the International Study of Asthma and Allergies in Childhood (ISAAC). Thorax. 2007;62(9):-. doi:10.1136/thx.2006.070169 17504817 PMC2117323

[2] Rawshani A, Landin-Olsson M, Svensson AM, The incidence of diabetes among 0-34 year olds in Sweden: new data and better methods. Diabetologia. 2014;57(7):1375-1381. doi:10.1007/s00125-014-3225-9 24710965 PMC4052006

[3] Huang SW Asthma and diabetes. Lancet. 1999;354(9177):515. doi:10.1016/S0140-6736(05)75551-3 10465204

[4] Kero J, Gissler M, Hemminki E, Isolauri E Could T_H_1 and T_H_2 diseases coexist? evaluation of asthma incidence in children with coeliac disease, type 1 diabetes, or rheumatoid arthritis: a register study. J Allergy Clin Immunol. 2001;108(5):781-783. doi:10.1067/mai.2001.119557 11692104

[5] Yun HD, Knoebel E, Fenta Y, Asthma and proinflammatory conditions: a population-based retrospective matched cohort study. Mayo Clin Proc. 2012;87(10):953-960. doi:10.1016/j.mayocp.2012.05.020 22980164 PMC3538394

[6] Hsiao YT, Cheng WC, Liao WC, Type 1 diabetes and increased risk of subsequent asthma: a nationwide population-based cohort study. Medicine (Baltimore). 2015;94(36):e1466. doi:10.1097/MD.0000000000001466 26356702 PMC4616625

[7] Metsälä J, Lundqvist A, Virta LJ, The association between asthma and type 1 diabetes: a paediatric case-cohort study in Finland, years 1981-2009. Int J Epidemiol. 2018;47(2):409-416. doi:10.1093/ije/dyx245 29211844

[8] Hemminki K, Li X, Sundquist J, Sundquist K Subsequent autoimmune or related disease in asthma patients: clustering of diseases or medical care? Ann Epidemiol. 2010;20(3):217-222. doi:10.1016/j.annepidem.2009.11.007 20036578

[9] Simpson CR, Anderson WJA, Helms PJ, Coincidence of immune-mediated diseases driven by Th1 and Th2 subsets suggests a common aetiology: a population-based study using computerized general practice data. Clin Exp Allergy. 2002;32(1):37-42. doi:10.1046/j.0022-0477.2001.01250.x 12002734

[10] Sheikh A, Smeeth L, Hubbard R There is no evidence of an inverse relationship between T_H_2-mediated atopy and T_H_1-mediated autoimmune disorders: lack of support for the hygiene hypothesis. J Allergy Clin Immunol. 2003;111(1):131-135. doi:10.1067/mai.2003.8 12532108

[11] Stene LC, Nafstad P Relation between occurrence of type 1 diabetes and asthma. Lancet. 2001;357(9256):607-608. doi:10.1016/S0140-6736(00)04067-8 11558491

[12] Cardwell CR, Shields MD, Carson DJ, Patterson CC A meta-analysis of the association between childhood type 1 diabetes and atopic disease. Diabetes Care. 2003;26(9):2568-2574. doi:10.2337/diacare.26.9.2568 12941720

[13] Tirosh A, Mandel D, Mimouni FB, Zimlichman E, Shochat T, Kochba I Autoimmune diseases in asthma. Ann Intern Med. 2006;144(12):877-883. doi:10.7326/0003-4819-144-12-200606200-00004 16785476

[14] Rabin RL, Levinson AI The nexus between atopic disease and autoimmunity: a review of the epidemiological and mechanistic literature. Clin Exp Immunol. 2008;153(1):19-30. doi:10.1111/j.1365-2249.2008.03679.x 18505431 PMC2432093

[15] Black P Why is the prevalence of allergy and autoimmunity increasing? Trends Immunol. 2001;22(7):354-355. doi:10.1016/S1471-4906(01)01940-8 11460823

[16] Tedeschi A, Airaghi L Is affluence a risk factor for bronchial asthma and type 1 diabetes? Pediatr Allergy Immunol. 2006;17(7):533-537. doi:10.1111/j.1399-3038.2006.00445.x 17014630

[17] Tedeschi A, Airaghi L Common risk factors in type 1 diabetes and asthma. Lancet. 2001;357(9268):1622. doi:10.1016/S0140-6736(00)04764-4 11386320

[18] Saleh NM, Raj SM, Smyth DJ, Genetic association analyses of atopic illness and proinflammatory cytokine genes with type 1 diabetes. Diabetes Metab Res Rev. 2011;27(8):838-843. doi:10.1002/dmrr.1259 22069270 PMC3816329

[19] Ullemar V, Magnusson PKE, Lundholm C, Heritability and confirmation of genetic association studies for childhood asthma in twins. Allergy. 2016;71(2):230-238. doi:10.1111/all.12783 26786172

[20] Thomsen SF, van der Sluis S, Kyvik KO, Skytthe A, Backer V Estimates of asthma heritability in a large twin sample. Clin Exp Allergy. 2010;40(7):1054-1061. doi:10.1111/j.1365-2222.2010.03525.x 20528882

[21] Hyttinen V, Kaprio J, Kinnunen L, Koskenvuo M, Tuomilehto J Genetic liability of type 1 diabetes and the onset age among 22,650 young Finnish twin pairs: a nationwide follow-up study. Diabetes. 2003;52(4):1052-1055. doi:10.2337/diabetes.52.4.1052 12663480

[22] Kuo CF, Chou IJ, Grainge MJ, Familial aggregation and heritability of type 1 diabetes mellitus and coaggregation of chronic diseases in affected families. Clin Epidemiol. 2018;10:1447-1455. doi:10.2147/CLEP.S172207 30349392 PMC6186906

[23] Kyvik KO, Green A, Beck-Nielsen H Concordance rates of insulin dependent diabetes mellitus: a population based study of young Danish twins. BMJ. 1995;311(7010):913-917. doi:10.1136/bmj.311.7010.913 7580548 PMC2550917

[24] Hudson JI, Javaras KN, Laird NM, VanderWeele TJ, Pope HG Jr, Hernán MA A structural approach to the familial coaggregation of disorders. Epidemiology. 2008;19(3):431-439. doi:10.1097/EDE.0b013e31816a9de7 18379420

[25] Ludvigsson JF, Otterblad-Olausson P, Pettersson BU, Ekbom A The Swedish personal identity number: possibilities and pitfalls in healthcare and medical research. Eur J Epidemiol. 2009;24(11):659-667. doi:10.1007/s10654-009-9350-y 19504049 PMC2773709

[26] Ludvigsson JF, Almqvist C, Bonamy AKE, Registers of the Swedish total population and their use in medical research. Eur J Epidemiol. 2016;31(2):125-136. doi:10.1007/s10654-016-0117-y 26769609

[27] Ekbom A The Swedish Multi-generation Register. Methods Mol Biol. 2011;675:215-220. doi:10.1007/978-1-59745-423-0_10 20949391

[28] Yao S, Kuja-Halkola R, Thornton LM, Familial liability for eating disorders and suicide attempts: evidence from a population registry in Sweden. JAMA Psychiatry. 2016;73(3):284-291. doi:10.1001/jamapsychiatry.2015.2737 26764185

[29] Egbuonu F, Antonio FA, Edavalath M Effect of inhaled corticosteroids on glycemic status. Open Respir Med J. 2014;8(1):101-105. doi:10.2174/1874306401408010101 25674180 PMC4319206

[30] Hemminki K, Li X, Sundquist J, Sundquist K Familial association between type 1 diabetes and other autoimmune and related diseases. Diabetologia. 2009;52(9):1820-1828. doi:10.1007/s00125-009-1427-3 19543881

[31] Örtqvist AK, Lundholm C, Wettermark B, Ludvigsson JF, Ye W, Almqvist C Validation of asthma and eczema in population-based Swedish drug and patient registers. Pharmacoepidemiol Drug Saf. 2013;22(8):850-860. doi:10.1002/pds.3465 23754713

[32] Waernbaum I, Dahlquist G, Lind T Perinatal risk factors for type 1 diabetes revisited: a population-based register study. Diabetologia. 2019;62(7):1173-1184. doi:10.1007/s00125-019-4874-5 31041471 PMC6560018

[33] Patterson CC, Harjutsalo V, Rosenbauer J, Trends and cyclical variation in the incidence of childhood type 1 diabetes in 26 European centres in the 25 year period 1989-2013: a multicentre prospective registration study. Diabetologia. 2019;62(3):408-417. doi:10.1007/s00125-018-4763-3 30483858

[34] Ludvigsson JF, Ludvigsson J, Ekbom A, Montgomery SM Celiac disease and risk of subsequent type 1 diabetes: a general population cohort study of children and adolescents. Diabetes Care. 2006;29(11):2483-2488. doi:10.2337/dc06-0794 17065689

