# Supplementary material for: Familial Coaggregation of Asthma and Type 1 Diabetes in Children
Source: JAMA Netw Open. 2020 Mar 12;3(3):e200834. doi: 10.1001/jamanetworkopen.2020.0834 (PMC7068230; doi:10.1001/jamanetworkopen.2020.0834)
Supplement: Supplement. — eMethods. Detailed Methods eReferences. eTable 1. Asthma and Type 1 Diabetes in Each Sub-Cohort of Relatives eTable 2. Family-Level Bidirectional Associations Between Asthma and Type 1 Diabetes Across Different Types of Relatives in Children Born 2001-2013 eTable 3. Sensitivity Analyses for Within-Individual Analyses of the Associations Between Asthma and Type 1 Diabetes in Children Born 2001-2013, Assessed at End of Follow-Up and at Different Ages eTable 4. Sensitivity Analyses for Bidirectional Within-Individual Associations of the Risk (as Hazard Ratio) of Subsequent Disease (Asthma/Type 1 Diabetes) if Previous Exposure to the Other Disease (Type 1 Diabetes /Asthma) in Children Born 2001-2013, Followed From Birth Until Disease Onset, Death, Emigration, or 31st December 2015, Whichever Occurred First eTable 5. Sensitivity Analyses for Family-Level Bidirectional Associations Between Asthma and Type 1 Diabetes Across Full Siblings [file jamanetwopen-3-e200834-s001.pdf]

Supplementary Online Content

Smew AI, Lundholm C, Sävendahl L, Lichtenstein P, Almqvist C. Familial coaggregation of asthma and type 1 diabetes in children. *JAMA Netw Open*. 2020;3(3):e200834. doi:10.1001/jamanetworkopen.2020.0834

eMethods. Detailed Methods

eReferences.

eTable 1. Asthma and Type 1 Diabetes in Each Sub-Cohort of Relatives

eTable 2. Family-Level Bidirectional Associations Between Asthma and Type 1 Diabetes Across Different Types of Relatives in Children Born 2001-2013

eTable 3. Sensitivity Analyses for Within-Individual Analyses of the Associations Between Asthma and Type 1 Diabetes in Children Born 2001-2013, Assessed at End of Follow-Up and at Different Ages

eTable 4. Sensitivity Analyses for Bidirectional Within-Individual Associations of the Risk (as Hazard Ratio) of Subsequent Disease (Asthma/Type 1 Diabetes) if Previous Exposure to the Other Disease (Type 1 Diabetes /Asthma) in Children Born 2001-2013, Followed From Birth Until Disease Onset, Death, Emigration, or 31st December 2015, Whichever Occurred First

eTable 5. Sensitivity Analyses for Family-Level Bidirectional Associations Between Asthma and Type 1 Diabetes Across Full Siblings

This supplementary material has been provided by the authors to give readers additional information about their work.

© 2020 Smew AI et al. *JAMA Network Open.*

eMethods. Detailed Methods

Definitions of asthma and type 1 diabetes were based on a combination of diagnoses and dispensed medication prescriptions from both the National Patient Register (NPR), which contains information on all inpatient diagnoses since 1987 and specialist outpatient diagnoses with approximately 80% coverage since 2001 [1], and the Swedish Prescribed Drug Register (SPDR), which includes complete coverage of dispensed medication since July 2005 (eMethods).

Diagnoses followed the International Classification of Diseases and Related Health Problems, Tenth Revision (ICD-10) and medication was registered using Anatomical Therapeutic Chemical classification (ATC) codes. Data were available for NPR until December 31, 2013 and for SPDR until December 31, 2015.

According to the algorithm of a previous Swedish validation study [2], shown to correspond well to Swedish diagnostic guidelines, we defined asthma based on children fulfilling at least one of the following medication criteria:

a) Two or more dispenses of inhaled corticosteroids (ICS; R03BA) and/or leukotriene receptor antagonists (LTRA; RO3DC03) and/or fixed combinations of beta2-agonists and corticosteroids (R03AK), with at least two weeks between dispenses in children younger than 4.5 years and regardless of time between dispenses in children older than 4.5 years.

b) Three of more dispenses of the asthma medication listed in a) and/or short acting beta2-agonists (R03AC02, R03AC03, R03AC12, R03AC13) within a 12-month period.

Given the lower PPV in the validation study for children below 4.5 years, where the medication criteria reflects both asthma and wheeze, compared to above 4.5 years, children below 4.5 years were required to both fulfil the aforementioned medication criteria AND have an asthma diagnosis (ICD-10 J45-46) in the National Patient Register (NPR) in order to be defined as an asthma case. However, children older than 4.5 years were defined as asthmatic if they either fulfilled the medication criteria OR had a diagnosis.

For this reason, only children 4.5 years or older on December 31, 2013 (end of data availability from NPR) were followed until December 31, 2015, allowing for the possibility of asthma definition to be solely based on medication information from the Swedish Prescribed Drug Register. In contrast, follow-up ended on December 31, 2013 for children younger than 4.5 years on that date, since these younger children required both diagnosis and medication in order to be defined as having asthma.

Date of asthma onset was defined as diagnosis date or date of first prescription of any asthma medication, whichever happened first.

Type 1 diabetes was defined as having any diagnosis of type 1 diabetes (ICD-10 E10) or any prescription of insulin (ATC A10A), to allow for identification of cases during the whole period of data availability up to 2015. Type 1 diabetes has a unique ICD-10 code and the use of insulin prescription as a proxy has been validated [3]. Date of type 1 diabetes onset was set as date of diagnosis or, if the individual was missing a diagnosis, as one week before dispense of the first insulin prescription.

© 2020 Smew AI et al. *JAMA Network Open.*

eReferences

1. Ludvigsson JF, Andersson E, Ekbom A, et al. External review and validation of the Swedish national inpatient register. *BMC Public Health*. 2011;11(1):450.

2. Örtqvist AK, Lundholm C, Wettermark B, Ludvigsson JF, Ye W, Almqvist C. Validation of asthma and eczema in population-based Swedish drug and patient registers. *Pharmacoepidemiol Drug Saf.* 2013;22(8):850-860.

3. Rawshani A, Landin-Olsson M, Svensson AM, et al. The incidence of diabetes among 0-34 year olds in Sweden: New data and better methods. *Diabetologia*. 2014;57(7):1375-1381.

© 2020 Smew AI et al. *JAMA Network Open.*

eTable 1. Asthma and type 1 diabetes in each sub-cohort of relatives.

Type of relative	Degree of shared segregating genes	Unique individuals, No.	Pairs in analyses, No.	Asthma, No. (%)	T1D, No. (%)	Both asthma and T1D, No. (%)
Full siblings	50 %	835 412	1 083 788	103 886 (9.6)	3077 (0.3)	393 (0.04)
Maternal half-siblings	25 %	67 611	85 618	9338 (10.9)	234 (0.3)	35 (0.04)
Paternal half-siblings	25 %	63 955	83 582	8965 (10.7)	234 (0.3)	36 (0.04)
Full cousins	12.5 %	837 024	2 581 136	251 213 (9.7)	7813 (0.3)	953 (0.04)
Half-cousins	6.25 %	232 777	876 402	95 072 (10.9)	2786 (0.3)	354 (0.04)

Abbreviations: T1D, type 1 diabetes.

© 2020 Smew AI et al. *JAMA Network Open.*

eTable 2. Family-level bidirectional associations[a] between asthma and type 1 diabetes across different types of relatives in children born 2001-2013.

**Asthma in relatives of individuals with T1D**								
	**Relatives of individuals with T1D**		**Relatives of individuals without T1D**					
	**Total, No.**	**Asthma, No. (%)**	**Total, No.**	**Asthma, No. (%)**	**Crude OR (95% CI)**	**Adjusted OR[b] (95% CI)**	**Adjusted OR[c] (95% CI)**	**Adjusted OR[d] (95% CI)**
Full siblings	3078	365 (11.86)	1 080 711	103 521 (9.58)	1.27 (1.13-1.42)	1.25 (1.12-1.40)	1.19 (1.06-1.34)	1.13 (1.01-1.28)
Maternal half-siblings	234	19 (8.12)	85 384	9319 (10.91)	0.72 (0.45-1.16)	0.72 (0.45-1.16)	0.82 (0.51-1.33)	0.79 (0.49-1.29)
Paternal half-siblings	234	28 (11.97)	83 348	8937 (10.72)	1.13 (0.74-1.73)	1.13 (0.74-1.73)	1.31 (0.85-2.01)	1.27 (0.82-1.97)
Full cousins	7813	816 (10.44)	2 573 323	250 397 (9.73)	1.08 (1.00-1.17)	1.08 (1.00-1.17)	1.05 (0.97-1.13)	1.04 (0.96-1.12)
Half-cousins	2786	335 (12.02)	873 616	94 737 (10.84)	1.12 (0.96-1.31)	1.12 (0.96-1.31)	1.13 (0.96-1.32)	1.13 (0.96-1.32)
**T1D in relatives of individuals with asthma**								
	**Relatives of individuals with asthma**		**Relatives of individuals without asthma**					
	**Total, No.**	**T1D, No. (%)**	**Total, No.**	**T1D, No. (%)**	**Crude OR (95% CI)**	**Adjusted OR[b] (95% CI)**	**Adjusted OR[c] (95% CI)**	**Adjusted OR[d] (95% CI)**
Full siblings	103 886	365 (0.35)	979 902	2712 (0.28)	1.27 (1.13-1.42)	1.21 (1.08-1.36)	1.19 (1.06-1.33)	1.16 (1.03-1.30)
Maternal half-siblings	9338	19 (0.20)	76 280	215 (0.28)	0.72 (0.45-1.16)	0.70 (0.43-1.13)	0.79 (0.49-1.27)	0.77 (0.48-1.25)
Paternal half-siblings	8965	28 (0.31)	74 617	206 (0.28)	1.13 (0.74-1.73)	1.11 (0.73-1.70)	1.33 (0.87-2.03)	1.35 (0.81-2.02)
Full cousins	251 213	816 (0.32)	2 329 923	6997 (0.30)	1.08 (1.00-1.17)	1.07 (0.99-1.15)	1.05 (0.97-1.13)	1.05 (0.97-1.13)
Half-cousins	95 072	335 (0.35)	781 330	2451 (0.31)	1.12 (0.97-1.31)	1.12 (0.96-1.30)	1.13 (0.97-1.31)	1.12 (0.97-1.31)

Abbreviations: OR, odds ratio; T1D, type 1 diabetes.
[a] The models estimate risk of asthma or T1D in relatives of individuals with T1D or asthma, using a robust sandwich estimator to correct for non-independence in familial clustering.
[b] Model adjusted for asthma in relative when estimating risk of T1D in relative, and T1D in relative when estimating risk of asthma in relative.
[c] Model adjusted for sex and date of birth of relative.
[d] Model adjusted for both [b] and [c].

© 2020 Smew AI, et al. *JAMA Network Open.*

eTable 3. Sensitivity analyses for within-individual analyses of the associations between asthma and type 1 diabetes in children born 2001-2013, assessed at end of follow-up and at different ages.

	Total, No.	Crude OR (95% CI)	Adjusted OR[a] (95% CI)
**S1[b]**			
End of follow-up	1 284 664	1.15 (1.04-1.26)	1.15 (1.04-1.26)
At age 5 y	954 539	1.30 (1.11-1.54)	1.29 (1.10-1.52)
At age 6 y	845 561	1.34 (1.17-1.55)	1.34 (1.16-1.54)
At age 7 y	742 237	1.43 (1.25-1.63)	1.42 (1.25-1.62)
At age 8 y	641 069	1.42 (1.26-1.61)	1.42 (1.25-1.60)
**S2[c]**			
End of follow-up	1 284 748	1.16 (1.04-1.29)	1.16 (1.04-1.29)
At age 5 y	954 592	1.25 (1.06-1.48)	1.25 (1.05-1.48)
At age 6 y	845 600	1.26 (1.08-1.46)	1.26 (1.09-1.46)
At age 7 y	742 264	1.32 (1.15-1.51)	1.32 (1.15-1.52)
At age 8 y	641 083	1.31 (1.15-1.49)	1.31 (1.15-1.49)
**S3[d]**			
End of follow-up	1 284 748	1.14 (1.04-1.26)	1.14 (1.04-1.26)
At age 5 y	954 601	1.32 (1.12-1.55)	1.30 (1.11-1.54)
At age 6 y	845 614	1.37 (1.19-1.58)	1.36 (1.18-1.57)
At age 7 y	742 285	1.43 (1.25-1.63)	1.41 (1.24-1.61)
At age 8 y	641 113	1.43 (1.26-1.62)	1.40 (1.24-1.59)
**S4[e]**			
End of follow-up	871 521	1.29 (1.11-1.50)	1.30 (1.12-1.51)
At age 5 y	549 282	1.52 (1.25-1.85)	1.53 (1.26-1.87)
At age 6 y	441 542	1.72 (1.44-2.04)	1.73 (1.45-2.06)
At age 7 y	339 337	1.96 (1.66-2.31)	1.98 (1.68-2.34)
At age 8 y	239 200	2.19 (1.86-2.58)	2.17 (1.84-2.56)

© 2020 Smew AI, et al. *JAMA Network Open.*

Abbreviations: OR, odds ratio; T1D, type 1 diabetes .
[a] Analyses adjusted for sex and date of birth.
[b] S1. Stricter definition of T1D, excluding children diagnosed prior to 1 year of age.
[c] S2. Stricter definition of T1D, requiring diagnosis in National Patient Register.
[d] S3. Stricter definition T1D, requiring dispense of insulin prescription in Swedish Prescribed Drug Register.
[e] S4. Restricted cohort of children born January 1, 2005 - December 31, 2013.

© 2020 Smew AI, et al. *JAMA Network Open.*

eTable 4. Sensitivity analyses for bidirectional within-individual associations of the risk (as hazard ratio) of subsequent disease (asthma/type 1 diabetes) if previous exposure to the other disease (type 1 diabetes /asthma) in children born 2001-2013, followed from birth until disease onset, death, emigration, or 31st December 2015, whichever occurred first.

	Total, No.	Previous disease, No.	Subsequent disease, No.	Mean length of follow-up (SD), y	Crude HR (95% CI)	Adjusted HR[a] (95% CI)
**S1[b]**						
Subsequent asthma	1 284 664	2994	91	6.6 (4.6)	0.92 (0.75-1.13)	0.91 (0.74-1.12)
Subsequent T1D	1 284 664	121 386	390	7.4 (4.1)	1.15 (1.05-1.26)	1.16 (1.06-1.28)
**S2[c]**						
Subsequent asthma	1 284 748	2568	95	6.6 (4.6)	0.93 (0.76-1.14)	0.93 (0.76-1.13)
Subsequent T1D	1 284 748	121 390	305	7.4 (4.1)	1.17 (1.06-1.30)	1.20 (1.09-1.34)
**S3[d]**						
Subsequent asthma	1 284 748	2946	92	6.6 (4.6)	0.94 (0.77-1.15)	0.93 (0.76-1.14)
Subsequent T1D	1 284 748	121 390	378	7.4 (4.1)	1.14 (1.04-1.25)	1.15 (1.05-1.26)
**S4[e]**						
Subsequent asthma	871 521	1166	28	4.5 (3.3)	0.93 (0.65-1.35)	0.94 (0.65-1.37)
Subsequent T1D	871 521	70 567	171	5.6 (2.7)	1.26 (1.09-1.45)	1.26 (1.10-1.45)

Abbreviations: HR, hazard ratio; T1D, type 1 diabetes.
[a] Adjusted for sex and date of birth.
[b] S1. Stricter definition of T1D, excluding children diagnosed prior to 1 year of age.
[c] S2. Stricter definition of T1D, requiring diagnosis in National Patient Register.
[d] S3. Stricter definition T1D, requiring dispense of insulin prescription in Swedish Prescribed Drug Register.
[e] S4. Restricted cohort of children born January 1, 2005 - December 31, 2013.

© 2020 Smew AI, et al. *JAMA Network Open.*

eTable 5. Sensitivity analyses for family-level bidirectional associations[a] between asthma and type 1 diabetes across full siblings.

Asthma in full siblings of individuals with T1D								
	Full siblings of individuals with T1D		Full siblings of individuals without T1D					
	Total, No.	Asthma, No. (%)	Total, No.	Asthma, No. (%)	Crude OR (95% CI)	Adjusted OR[b] (95% CI)	Adjusted OR[c] (95% CI)	Adjusted OR[d] (95% CI)
S1[e]	2664	350 (11.69)	1 080 640	103 512 (9.58)	1.25 (1.11-1.40)	1.23 (1.10-1.38)	1.17 (1.04-1.31)	1.12 (0.99-1.26)
S2[f]	2430	287 (11.81)	1 081 358	103 599 (9.58)	1.25 (1.11-1.43)	1.24 (1.10-1.41)	1.18 (1.04-1.33)	1.11 (0.98-1.27)
S3[g]	2942	349 (11.86)	1 080 846	103 537 (9.58)	1.27 (1.13-1.43)	1.25 (1.12-1.40)	1.19 (1.06-1.34)	1.14 (1.01-1.28)
S4[h]	969	88 (9.08)	572 735	46 614 (8.14)	1.13 (0.90-1.42)	1.12 (0.89-1.40)	1.14 (0.91-1.44)	1.07 (0.84-1.36)
T1D in full siblings of individuals with asthma								
	Full siblings of individuals with asthma		Full siblings of individuals without asthma					
	Total, No.	T1D, No. (%)	Total, No.	T1D, No. (%)	Crude OR (95% CI)	Adjusted OR[b] (95% CI)	Adjusted OR[c] (95% CI)	Adjusted OR[d] (95% CI)
S1[e]	103 862	350 (0.34)	979 722	2644 (0.27)	1.25 (1.11-1.40)	1.19 (1.06-1.34)	1.17 (1.04-1.31)	1.14 (1.01-1.28)
S2[f]	103 886	287 (0.28)	979 902	2143 (0.22)	1.26 (1.12-1.43)	1.20 (1.05-1.36)	1.17 (1.03-1.33)	1.14 (1.00-1.30)
S3[g]	103 886	349 (0.34)	979 902	2593 (0.26)	1.27 (1.13-1.43)	1.21 (1.08-1.37)	1.19 (1.06-1.34)	1.16 (1.03-1.31)
S4[h]	46 702	88 (0.19)	527 002	881 (0.17)	1.13 (0.90-1.42)	1.06 (0.84-1.35)	1.09 (0.87-1.37)	1.06 (0.84-1.34)

Abbreviations: OR, odds ratio; T1D, type 1 diabetes.
[a] The models estimate risk of asthma or T1D in relatives of individuals with T1D or asthma, using a robust sandwich estimator to correct for non-independence in familial clustering.
[b] Adjusted for asthma in relative when estimating risk of T1D in relative, and T1D in relative when estimating risk of asthma in relative.
[c] Adjusted for sex and date of birth of relative.
[d] Adjusted for both [b] and [c].
[e] S1. Stricter definition of T1D, excluding children diagnosed prior to 1 year of age.
[f] S2. Stricter definition of T1D, based on diagnosis in National Patient Register.
[g] S3. Stricter definition T1D, based on dispense of insulin prescription in Swedish Prescribed Drug Register.
[h] S4. Restricted cohort of children born January 1, 2005 - December 31, 2013.

© 2020 Smew AI, et al. *JAMA Network Open.*
